# Transplantation of EPCs overexpressing PDGFR-β promotes vascular repair in the early phase after vascular injury

**DOI:** 10.1186/s12872-016-0353-9

**Published:** 2016-09-13

**Authors:** Hang Wang, Yang-Guang Yin, Hao Huang, Xiao-Hui Zhao, Jie Yu, Qiang Wang, Wei Li, Ke-Yin Cai, Shi-Fang Ding

**Affiliations:** 1 Cadre Ward Two, Wuhan General Hospital of Guangzhou Military Command, Wuhan, 430070 China; 2 Intensive Care Unit, The sixth people’s hospital of Chongqing, Nan’an District, Chongqing, 400060 China; 3 Clinic center, Shenzhen Hornetcorn Biotechnology Company, Ltd, Shenzhen, 518400 China; 4 Institute of Cardiovascular Science, Xinqiao Hospital, Third Military Medical University, Chongqing, 400037 China; 5 Institute of Cardiovascular Science, Wuhan General Hospital of Guangzhou Military Command, Wuhan, 430070 China

**Keywords:** EPCs, PDGFR-β, Reendothelialization, Neointima, Gene therapy

## Abstract

**Background:**

Endothelial progenitor cells (EPCs) play important roles in the regeneration of the vascular endothelial cells (ECs). Platelet-derived growth factor receptor (PDGFR)-β is known to contribute to proliferation, migration, and angiogenesis of EPCs, this study aims to investigate effects of transplantation of EPCs overexpressing PDGFR-β on vascular regeneration.

**Methods:**

We transplanted genetically modified EPCs overexpressing PDGFR-β into a mouse model with carotid artery injury. After 3 days of EPCs transplantation, the enhanced green fluorescent protein (EGFP)-expressing cells were found at the injury site and the lining of the lumen by laser scanning confocal microscope (LSCM). At 4, 7, and 14 days of the carotid artery injury, reendothelialization was evaluated by Evans Blue staining. Neointima formation was evaluated at day 14 with hematoxylin and eosin (HE) staining by calculating the neointimal area, medial area, and neointimal/media (NI/M) ratio. Intimal cell apoptosis was evaluated using TUNEL assay. Then we tested whether PDGF-BB-induced VSMC migration and PDGF-BB’s function in reducing VSMC apoptosis can be attenuated by EPCs overexpressing PDGFR-β in a transwell co-culture system.

**Results:**

Our results showed that EPCs overexpressing PDGFR-β accelerates reendothelialization and mitigates neointimal formation at 14 days after injury. Moreover, we found that there is great possibility that EPCs overexpressing PDGFR-β enhanc VSMC apoptosis and suppress VSMC migration by competitive consumption of PDGF-BB in the early phase after carotid artery injury in mice.

**Conclusions:**

We report the first in vivo and in vitro evidence that transplantation of genetically modified EPC can have a combined effect of both amplifying the reendothelialization capacity of EPCs and inhibiting neointima formation so as to facilitate better inhibition of adverse remodeling after vascular injury.

## Background

The normal arterial vessel wall is mostly composed of endothelial cells (ECs), vascular smooth muscle cells (VSMCs), and macrophages. Endothelial impairment is believed to be a major contributor to atherosclerosis and restenosis after percutaneous coronary intervention (PCI) [1, 2]. Reendothelialization can effectively inhibit VSMC migration and proliferation and decrease neointimal thickening [3]. Therefore, acceleration of reendothelialization is of special interest with regard to reducing neointima formation to prevent postangioplasty restenosis and development of atherosclerosis.

Endothelial progenitor cells (EPCs) include cells in multiple stages from mother cells to mature ECs. Both early EPCs that can repair the blood vessels and the late EPCs that have strong proliferation ability are involved in angiogenesis. Indeed, the numbers of EPCs in a patient with atherosclerotic vascular disease, who needs endothelial repair, are much lower than that in a normal person [4–6]. Recently, several studies showed that EPCs can be recruited to the sites of endothelial injury, be differentiated into mature ECs, and can play important roles in reendothelialization after vascular injury [7–10]. Platelet-derived growth factor (PDGF) can enhance VSMC function and injury-induced neointima formation. PDGF-BB gene knockout mice show pathological defects such as heart and blood vessel dilations, proving that PDGF-BB plays a vital role in the establishment of the circulatory system in the body [11]. Recently, it was reported that PDGF-BB was locally produced by injured arteries, and it contributed to the promotion of migration, proliferation, and neointima formation of local VSMCs for participation in vascular repair/remodeling in human and animal vascular injury models [12]. However, inhibition of PDGF-BB signaling has been shown to reduce neointima formation and inhibit vascular repair/remodeling after angioplasty [13–15]. Our previous study also showed that overexpression of PDGF-receptor (PDGFR)-β promoted PDGF-BB-induced proliferation, migration, and angiogenesis of EPCs [16].

Based on known knowledge regarding PDGF-BB and PDGFR-β on the biological functions of VSMCs and EPCs, we propose that EPCs overexpressing PDGFR-β may have effects on reendothelialization during arterial repairment after injury. To test this, we transplanted genetically modified EPCs overexpressing PDGFR-β into a mouse model with carotid artery injury and evaluated whether locally released PDGF-BB can stimulate homing of the transplanted EPCs to the site of endothelial injury and improve their biological functions. We further investigated whether the homed EPCs overexpressing PDGFR-β can compete for the locally produced PDGF-BB produced by injured arteries with the VSMCs to inhibit the local VSMC migration, proliferation, and neointima formation. Our results showed that transplantation of genetically modified EPC may have a combined effect of both amplifying the reendothelialization capacity of EPCs for repairing injured arteries as well as inhibiting the capacity of EPCs in neointima formation so as to facilitate better inhibition of adverse remodeling after vascular injury.

## Methods

### Animals and protocols

All procedures were performed in compliance with the Ethic Committee of Third Military Medical University and the National Institute of Health Guide for the Care and Use of Laboratory. Animals Male C57BL/6 mice (weight: 25–30 g, age: 6–8 weeks) were obtained from the Laboratory Animal Center at the Third Military Medical University (Chongqing, China). Mice were firstly anaesthetized by 40 mg/kg sodium pentobarbital (Sigma-Aldrich, St Lois, MO, USA) via iintraperitoneal (IP) injection. The extent of anaesthesia was assessed by mouse’s reaction to the toe pinching during the splenectomy or the carotid artery injury surgery. Then they received 3 mg/kg ketorolac tromethamine (Newtime, Shandong, China) by per os(PO) to minimize the postoperative pain. At last all mice were euthanized by IP injection of 240 mg/kg sodium pentobarbital.

### Splenectomy

Splenectomy was performed as described in our previous study [17]. Vessels of the mice were carefully ligated using 6–0 silk ligatures via a lateral incision of the left abdomen, followed by ablation of the spleen. The abdomen was immediately closed layer by layer with single sutures using 6–0 silk. The mice were allowed to recover for 14 days after which the carotid arterial injury was induced.

### EPC culturing and characterization

Culturing and characterization of mouse spleen-derived EPCs was performed as described previously [16, 18]. To determine the endothelial phenotype of EPCs, the cells were incubated with 2.4 μg/mL acLDL-DiI (Invitrogen, CA, USA) for 4 h, fixed with 4 % paraformaldehyde (PFA), and then incubated with 10 μg/mL FITC-UEA-1 (Sigma-Aldrich, St. Louis, MO, USA) for 1 h. The cells positive for both acLDL-DiI and UEA-1 were identified as differentiating EPCs. In addition, the phenotypes of EPCs were evaluated by flow cytometry (FCM). Cells (1 × 106) were incubated with the following monoclonal antibodies: PE-conjugated anti-VEGFR-2 (eBiosciences, San Diego, CA, USA), FITC-conjugated anti-Sca-1 (abCAM, Cambridge, MA, USA), or their corresponding isotype controls (eBiosciences).

### EPCs gene transfer

The plasmids pEGFP-N2 and pEGFP-N2-PDGFR-β were kindly provided by Dr. Shangcheng Xu at the Third Military Medical University. Transfection was performed, as described previously [16], with the Lipofectamine™ 2000 reagent (Invitrogen, Shanghai, China), according to the manufacturer’s instruction. The EPCs of the pEGFP-N2 or pEGFP-N2-PDGFR-β groups were collected 24 h after transfection.

### VSMC culturing and characterization

VSMCs were isolated from the thoracic aorta of mice through explantation and cultured in Dulbecco’s Modified Eagle Medium: nutrient mixture F-12 (DMEM/F-12) culture medium (Gibco BRL, NY, USA) supplemented with 20 % fetal calf serum (FCS, Gibco BRL, NY, USA), 100 U/mL penicillin, and 100 U/mL streptomycin. Cells were kept at 37 °C in a 5 % CO_2_ atmosphere. Cultures were confirmed by smooth muscle α-actin (SMαA, NeoMarkers, CA, USA). Cells from 3 to 6 passages were used for all experiments.

### Carotid artery injury model and EPC transplantation

Injury of carotid artery was induced 14 days after splenectomy, as described in our previous study [19]. Briefly, the bifurcation of the left carotid artery was exposed through a midline incision on the ventral side of the neck. A 6–0 silk slipknot was placed around the common carotid artery and the internal carotid artery to block their blood flow. Two ligatures were placed proximally and distally around the external carotid artery, and the distal ligature was then tied off. A tailored hook made from a syringe (1 mL) was used to place the silk around the external and the internal carotid arteries. An incision was made between the two ligatures to introduce the denudation device. The tailored flexible wire (0.014-in. diameter, the tip of the wire was relatively thinner) was introduced into the common carotid artery. The endothelium was denuded by passing the wire back and forth through the vessel three times. After removal of the wire, the proximal ligature of the external carotid artery was tied off. The slipknots were removed, and the blood flow was restored. The skin incision was sutured with 6–0 silk. Then, the mice were administered 200 μL saline alone or 200 μL saline containing enhanced green fluorescent protein (EGFP)-labeled EPCs (1 × 10^6^) or EPCs overexpressing PDGFR-β (1 × 10^6^) via tail vein injection directly after endothelial injury of the carotid artery and again after 24 h.

### EPC tracing in vivo

To observe whether the transfected EPCs were capable of homing to the site of injury, labeled pEGFP-N2-EPCs and pEGFP-N2- PDGFR-β-EPCs (1 × 10^6^) were incubated with 2.4 μg/ml acLDL-DiI (Invitrogen, CA, USA) for 1 h. Then the cells were washed with PBS 3 times and injected into the mice’ tail vein in 200 μL saline after induction of arterial injury and again after 24 h. 7 days later, EPC tracking and immunohistochemistry were performed. Images of the stained cells were obtained by a fluorescence microscope (Leica).

### RNA extraction and semi-quantitative reverse transcription-polymerase chain reaction (RT-PCR)

Total RNA was extracted from the arteries by using RNAout (Tianenze Biotech, Beijing, China), and the cDNA was obtained through RT-PCR with the PrimeScript™ RT Reagent Kit (Takara, Biotechnology, Dalian, China) by using total RNA as a template, followed by amplification. The primers used were as follows: PDGFR-β (sense) 5′-CCGGCGCTGGCGAGTTAGTTT-3′, (antisense) 5′-ACACCTACTTTTGAGGTCTCTGCAGG-3′; product length 296 bp. PDGF-BB (sense) 5′-TGCTGAGCGACCACTCCATC-3′, (antisense) 5′-TGTGCTCGGGTCATGTTCAAG-3′; product length 109 bp. Glyceraldehyde 3-phosphate dehydrogenase (GAPDH) (sense) 5′- AACTTTGGCATTGTGGAAGGGCTC-3′, (antisense) 5′- ACCCTGTTGCTGTAGCCGTATTCA-3′; product length 473 bp. All primers were obtained from Invitrogen (Shanghai, China).

### Western blotting

The protein concentration of tissue lysates was estimated by the Bradford method, and the proteins were transferred onto polyvinylidene fluoride (PVDF) membranes. The membranes were blocked with 5 % non-fat milk, probed with anti-PDGFR-β (Abcam, USA), anti-PDGF-BB (Santa Cruz Biotechnology, USA), and anti-GAPDH (Cell Signaling Biotechnology, Beverly, MA, USA), followed by staining with horseradish peroxidase-coupled secondary antibodies. The protein bands were visualized by enhanced chemiluminescence (Amersham Pharmacia Biotech, UK) and quantified by using the Quantity One software (Bio-Rad, Hercules, USA).

### Immunofluorescence

Before and 7 days after the carotid artery injury, the carotid arteries were snap-frozen in liquid nitrogen in optimal cutting temperature (OCT)-embedding medium and stored at −80 °C. Six cross-sections were cut (5-μm thickness) from the approximate middle portion of the artery and used for the detection of PDGF-BB by immunofluorescence. For fluorescence staining, the sections were first incubated with an anti-PDGF-BB primary mAb (1:100) and then with a FITC-labeled secondary antibody (Beyotime, Shanghai, China). Images of the sections were obtained by a laser scanning confocal microscope (LSCM; Leica).

### Measurement of reendothelialization

After 4, 7, and 14 days of the carotid artery injury, endothelial regeneration was evaluated by staining the denuded areas by injecting 200 μL of 5 % Evans Blue dye with saline via the tail vein into the heart. The left common carotid artery was then harvested 5 mm away from the carotid bifurcation. The reendothelialized area appeared white in color (unstained), whereas the non-endothelialized lesions appeared blue (stained). The unstained-areas (in white) and the total carotid artery areas were measured. The ratio of reendothelialized areas (unstained area) versus the total carotid artery area were calculated.

### Assessment of neointimal and medial areas

For histological analysis, hematoxylin and eosin (HE) staining was performed, according to the standard protocols; three sections taken from the middle portion of each artery, 14 days after the carotid artery injury, were examined; and the neointimal area, medial area, and neointima/media (NI/M) ratio were calculated.

### Apoptosis assay in the intima

Seven days after the carotid artery injury, the carotid arteries were snap-frozen in liquid nitrogen in OCT-embedding medium and stored at −80 °C. Six cross-sections (5-μm thick), cut from the approximate middle portion of the artery, were used for the detection of intimal apoptotic cells by immunofluorescence for terminal deoxynucleotidyl transferase dUTP nick-end labeling (TUNEL) by using the in situ Cell Death Detection Kit (Roche), according to the manufacturer’s instructions, and then SmαA staining of VSMCs, DAPI staining of the nuclei of cells was performed. After the fluorescence staining, the numbers of TUNEL-positive and-negative nuclei were counted in five different high-power fields (HPFs) in each section under the LSCM. Apoptosis activity was expressed in terms of TUNEL-labeling index, calculated by dividing the positively labeled cells by the total cell number.

### VSMCs and EPCs co-culture

**Fig. 1:**
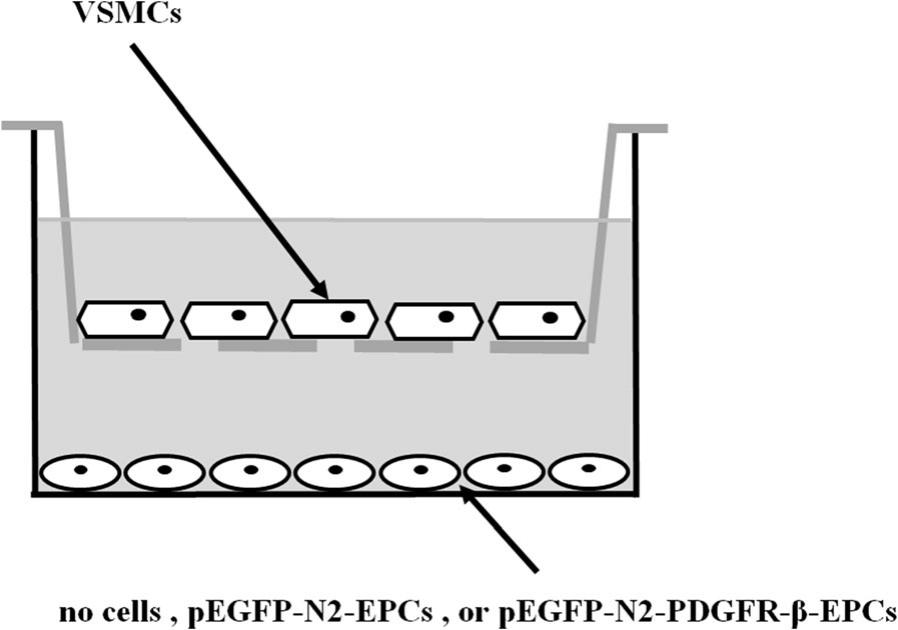
Illustration of the transwell co-culture system. The transwell consists of two chambers separated by a porous membrane. The VSMCs were placed on the membrane of the upper chamber while no cells, EPCs with the plasmid pEGFP-N2 transfection, or EPCs with the plasmid pEGFP-N2-PDGFR-β transfection were placed on the bottom of the lower chamber, respectively

For the settlement of the VSMC/EPC system, VSMCs were seeded on Transwell filters (4 μm pores or 8 μm pores, Corning Costar, USA). A total of 2.5 × 10^5^ EPCs were cultured and were transfected in a separate well, on the lower chamber of the system. After both types of cells reached confluency, the inserts with VSMCs were added to the wells where EPCs were cultured (resulting). Three groups of cells were VSMCs and EPCs co-cultured (Fig. 1): (1) control group: no cells were cultured on the lower chamber of the system; (2) pEGFP-N2 group : EPCs with the plasmid pEGFP-N2 transfection were cultured on the lower chamber of the system; (3) pEGFP-N2-PDGFR-β group : EPCs with the plasmid pEGFP-N2-PDGFR-β transfection were cultured on the lower chamber of the system.

### VSMC migration assay

The co-culture migration assay was examined using the VSMC/EPC system containing 8 μm polycarbonate filter inserts in 24-well plates. Three groups of cells were filled in various concentrations (0, 20, 40, 60, or 80 ng/mL) PDGF-BB respectively. After 12 h in culture, VSMCs on the bottom of the Transwell membrane were fixed with 4 % PFA at 37 °C for 20 min and stained with 1 % crystal violet at 37 °C for 5 min. The number of migrating cells on the bottom of the Transwell in 5 randomly HPFs (×200) was counted manually. Results were representative of three independent experiments.

### VSMC apoptosis assay

The co-culture apoptosis assay was identified by immunofluorescence for TUNEL by using the in situ Cell Death Detection Kit (Roche, USA), according to the manufacturer’s instructions. Briefly, the VSMC/EPC system containing 4 μm polycarbonate filter inserts in 24-well plates were used. Three groups of cells were treated with various concentrations (0, 20, 40, 60, or 80 ng/mL) PDGF-BB in DMEM/F-12 with 1 % FCS (apoptotic condition) for 72 h. VSMCs were fixed in 4 % PFA for 20 min and then treated with permeabilization solution (0.2 % Triton X-100 solution in PBS) for 5 min at room temperature. Labeling reactions were performed with 100 μL of reaction buffer for 60 min at 37 °C in a in the dark. DAPI staining of the nuclei of VSMCs was performed. After the fluorescence staining, the numbers of TUNEL-positive and-negative nuclei were counted in five HPFs (×200). Results were representative of three independent experiments. The TUNEL-labeling index was counted manually.

### Statistical analysis

Data from independent experiments were expressed as mean ± standard deviation (SD). Statistical analyses were performed with the SPSS 13.0 software (SPSS Inc, Chicago, USA). Comparisons between the groups were performed by two-tailed Student’s *t*-test or analysis of variance (ANOVA). Comparisons between multiple groups were tested by Multi-Way ANOVA. *P* < 0.05 was considered statistically significant.

## Results

### Overexpression of PDGFR-β in transfected EPCs

**Fig. 2:**
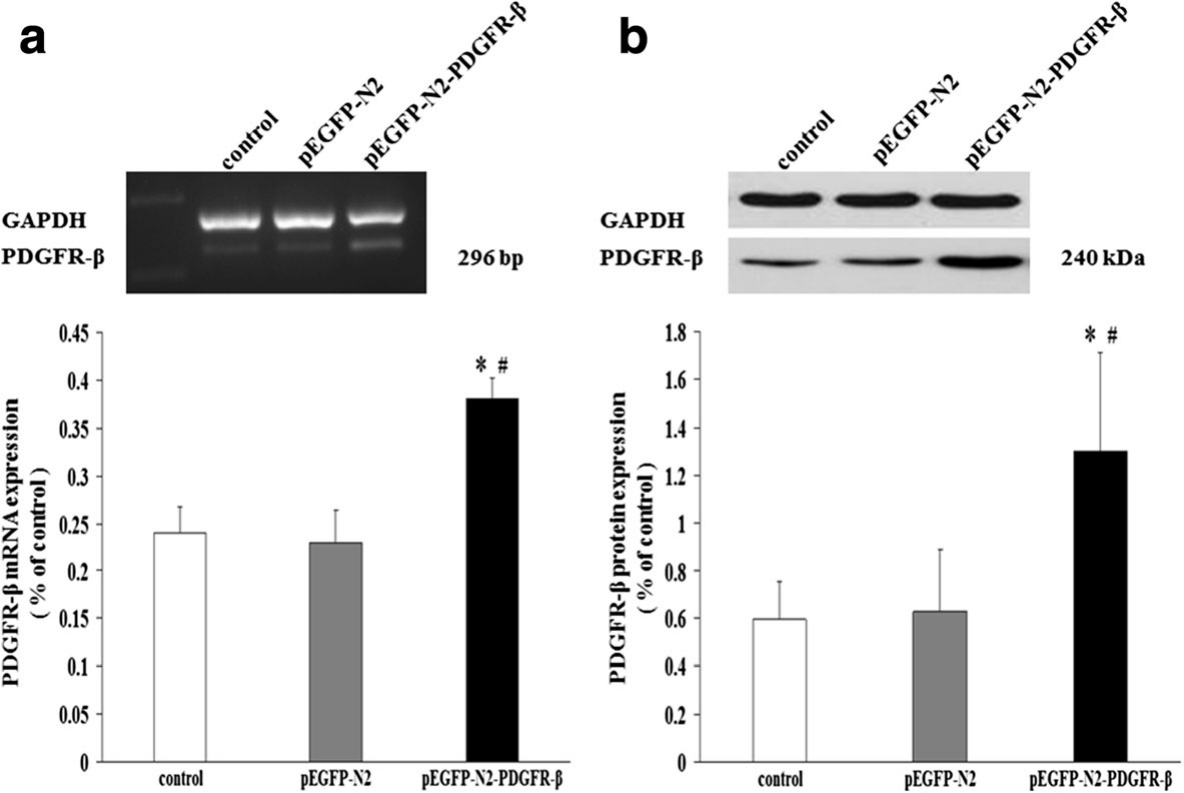
Overexpression of PDGFR-β in transfected EPCs. At 10 days after transfection, PDGFR-β mRNA level (**a**) and protein levels (**b**) in the pEGFP-N2-PDGFR-β group were significantly higher than those in the control group and pEGFP-N2 group.*P < 0.05 vs. control or ^#^ *P* < 0.05 vs. pEGFP-N2 (*n* = 3)

EPCs were transfected with either pEGFP-N2 or pEGFP-N2-PDGFR-β. After 10 days of transfection, the mRNA level of PDGFR-β in the pEGFP-N2-PDGFR-β group (0.38 ± 0.02) was significantly increased as compared to that in the non-transfected group (0.24 ± 0.03, *p* < 0.05) or the pEGFP-N2 group (0.23 ± 0.04, *p* < 0.05), as determined by semi-quantitative RT-PCR (Fig. 2a). Similarly, significantly increased protein level of PDGFR-β was also found in the pEGFP-N2-PDGFR-β group (1.30 ± 0.41) compared to that in the non-transfected group (0.60 ± 0.16, *p* < 0.05) and the pEGFP-N2 group (0.63 ± 0.26, *p* < 0.05), as determined by western blotting (Fig. 2b). Our results showed that this overexpression was maintained for at least 10 days.

### Expression of PDGF-BB after wire-mediated carotid artery injury in mice

**Fig. 3:**
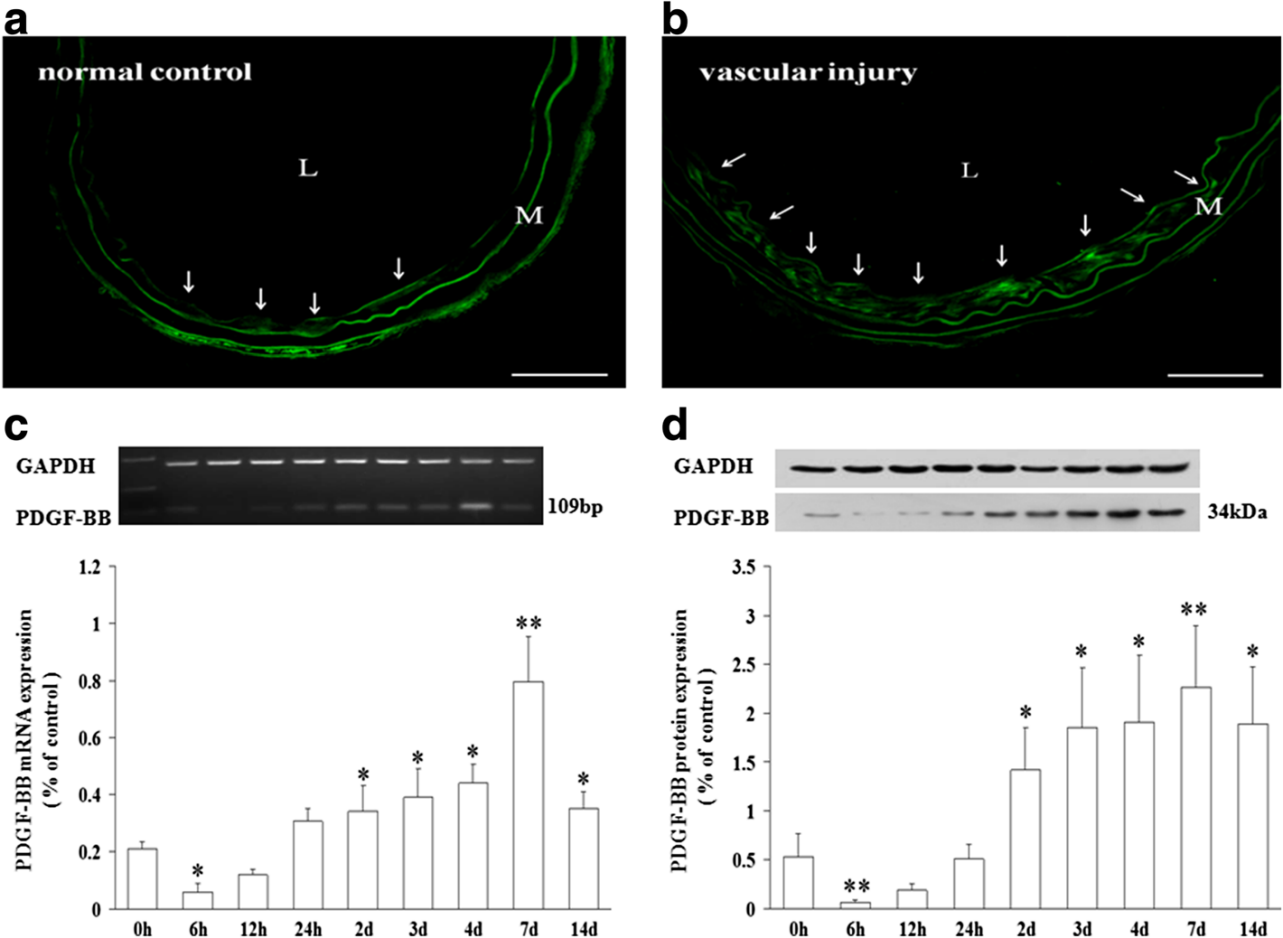
Comparison of expression of PDGF-BB in normal and vascular injured mice. Arrows indicate immunofluorescence staining for PDGF-BB (green) in normal (**a**) and wire-mediated carotid artery injury mice (**b**). **c** Semi-quantitative RT-PCR revealed that PDGF-BB was significantly enhanced at day 7. **d** Top: Representative images from western blotting. Bottom: Protein level of PDGF-BB, as assessed by densitometric analysis. *P < 0.05 vs. 0 h; **P < 0.01 vs. 0 h (*n* = 3). L, lumen; M, media. Scale bar = 50 μm

Localization of the PDGF-BB in injured vessels was investigated by immunofluorescence. PDGF-BB was rarely observed in uninjured carotids (Fig. 3a), but observed in the intima of local injured carotids at day 7 (Fig. 3b).

We analyzed the PDGF-BB expression during vascular injury following wire-mediated carotid artery injury in mice. As shown in Fig. 3c, the PDGF-BB mRNA expression was detected at low levels in uninjured control arteries (0 h), and started reducing rapidly at 6 h (3.51-fold). However, the PDGF-BB mRNA level was significantly enhanced at day 7 (3.80-fold) after the vascular injury, followed by a gradual decline at day 14 (1.67-fold).

The PDGF-BB protein expression was assessed by western blotting. The protein level started reducing rapidly at 6 h (7.80-fold) and 12 h (2.82-fold). However, the level began increasing gradually at day 2 (2.67-fold), with significant enhancement at day 7 (4.26-fold; Fig. 3d).

### Labeled EPCs were observed in injured artery

**Fig. 4:**
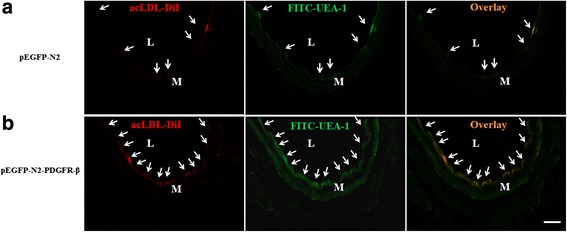
EPCs tracing in vivo. **a** Labeled pEGFP-N2-EPCs and **b** pEGFP-N2- PDGFR-β-EPCs were injected into the mice after vascular injury and attached to the vascular injury site on day 7. Arrows indicate EPCs (*n* = 5). L, lumen; M, media. Scale bar = 50 μm

After 7 days of EPC transplantation, acLDL-DiI-labeled EPCs were identified as red fluorescence cells (Fig. 4a, b). Labeled cells were seen lining the lumen that co-stained for endothelial markers FITC-UEA-1. No acLDL-DiI-labeled cells were identified in uninjured control arteries (data not shown).

### Transplantation of EPCs overexpressing PDGFR-β promoted reendothelialization

**Fig. 5:**
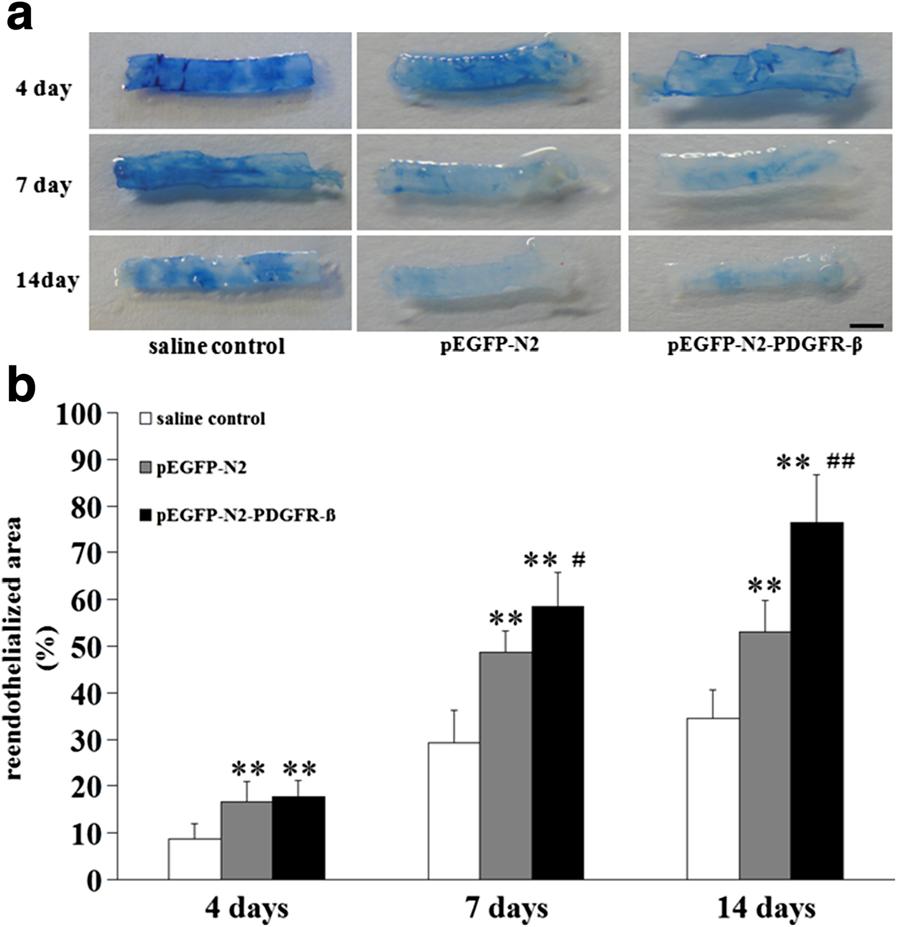
Reendothelialization of injured carotid arteries is promoted by EPC transplantation and further enhanced by transplantation with EPCs overexpressing PDGFR-β. **a** Representative images of reendothelialization at days 4, 7, and 14 after wire-mediated carotid artery injury in the saline control, pEGFP-N2, and pEGFP-N2-PDGFR-β groups. **b** Quantification of Evans blue staining showed that PDGFR-β overexpression accelerated reendothelialization at days 7 and 14 after wire-mediated carotid artery injury, while no difference was observed at day 4. ** *P* < 0.01 vs. saline control group; ^#^ *P* < 0.05 vs. pEGFP-N2 group; ^##^ *P* < 0.01 vs. pEGFP-N2 group (*n* = 8 per study group). Scale bar = 1 mm

To investigate whether PDGFR-β overexpression could accelerate reendothelialization, Evans Blue staining was performed (Fig. 5a). At day 4, the reendothelialized area in the pEGFP-N2-PDGFR-β-EPCs transplanted arteries (17.76 ± 3.35 %) was significantly larger than in the saline control group (8.83 ± 3.16 %, *p* < 0.01) but was not significantly larger (*P* > 0.05) than that in the pEGFP-N2-EPCs transplanted arteries (16.56 ± 4.46 %; Fig. 5b). At day 7, the reendothelialized area in the pEGFP-N2-PDGFR-β-EPCs transplanted arteries (58.55 ± 7.17 %) was significantly larger than that in the pEGFP-N2-EPCs transplanted arteries (48.62 ± 4.55 %; *p* < 0.05; Fig. 5b) and that in the saline control group (29.15 ± 7.07 %, *p* < 0.01). At day 14, the reendothelialized area in the pEGFP-N2-PDGFR-β-EPCs transplanted arteries (76.41 ± 10.16 %) was significantly larger than that in the pEGFP-N2-EPCs transplanted arteries (53.00 ± 7.98 %, *p* < 0.01) and that in the saline control group (34.6 ± 6.06 %, *p* < 0.01). These results demonstrate that reendothelialization of injured carotid arteries is promoted by EPC transplantation and further enhanced by transplantation with EPCs overexpressing PDGFR-β at days 7 and 14 after wire-mediated carotid artery injury.

### Transplantation of EPCs overexpressing PDGFR-β attenuated neointima formation

**Fig. 6:**
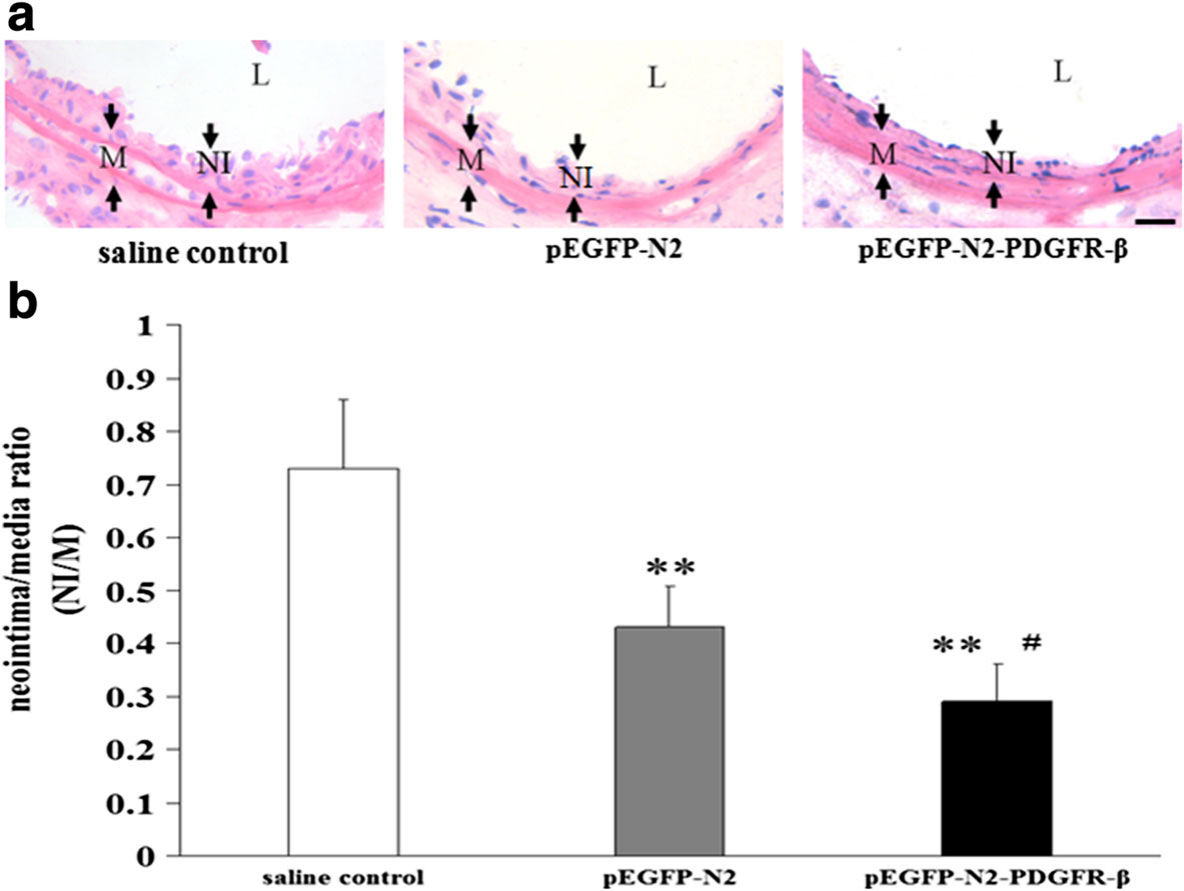
Transplantation of EPCs overexpressing PDGFR-β inhibited neointima formation at day 14 after carotid artery injury. **a** Representative photos of carotid artery were stained with HE. **b** Neointima/media ratios in the injured vessels of the saline-treated, pEGFP-N2-EPCs-transplanted, and pEGFP-N2-PDGFR-β-EPCs-transplanted groups. **P < 0.01 vs. saline control group; ^#^ *P* < 0.05 vs.pEGFP-N2-EPCs group (*n* = 12 per study group). L, lumen; M, media; NI, neointima. Scale bar = 50 μm

The effect of transplantation of EPCs overexpressing PDGFR-β on neointima formation was evaluated by HE staining at 14 days after the carotid injury. At day 14, a significant decrease (*P* < 0.05) in NI/M ratio was noted in the pEGFP-N2-PDGFR-β-EPCs group (0.29 ± 0.07) as compared with that in the pEGFP-N2-EPCs group (0.43 ± 0.08; Fig. 6a, b) and in the saline control group (0.73 ± 0.13, *p* < 0.01). Our results indicate that the transplantation of EPCs overexpressing PDGFR-β can inhibit neointima formation in the early phase after carotid artery injury.

### Transplantation of EPCs overexpressing PDGFR-β increased intima cells apoptosis

**Fig. 7:**
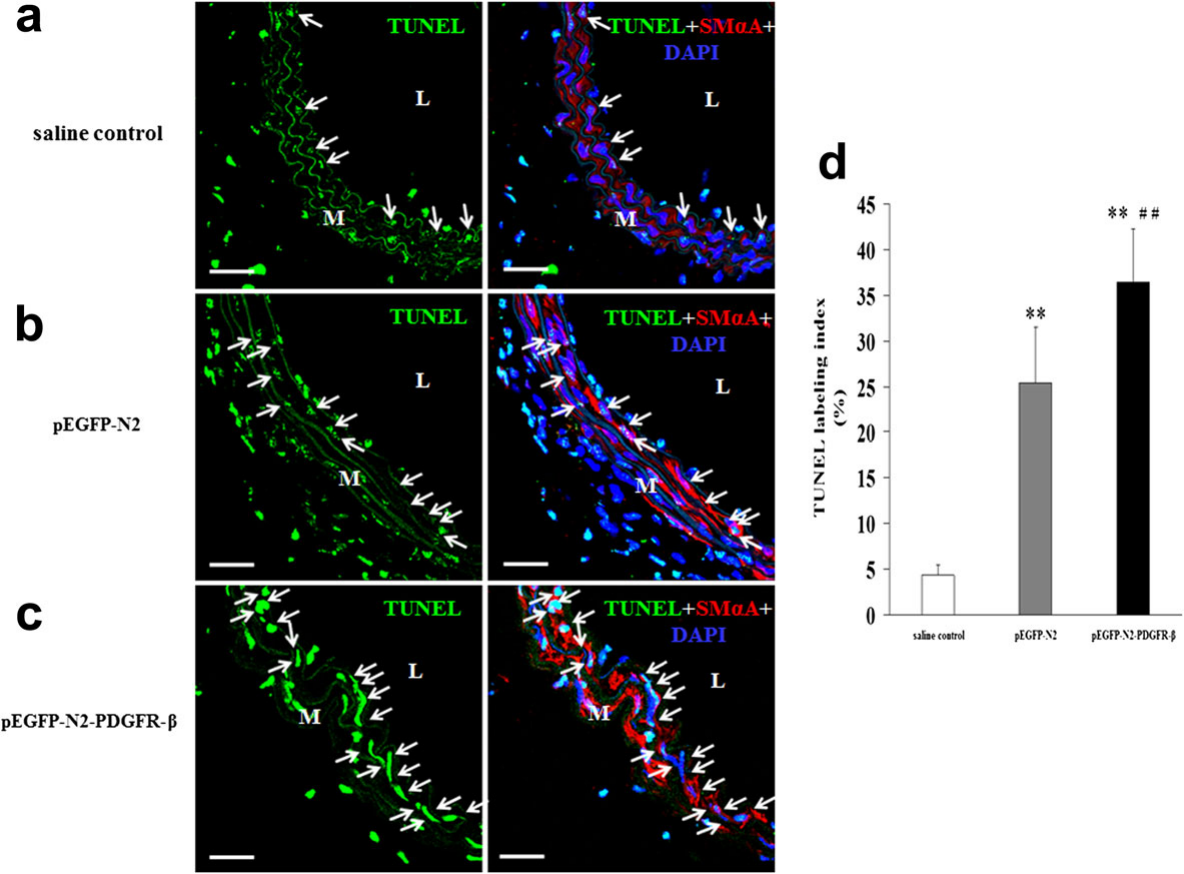
Transplantation of EPCs overexpressing PDGFR-β induces apoptosis of medial cells (VSMCs). Fluorescent TUNEL staining in injured arteries of the **a** saline control group, **b** pEGFP-N2-EPCs group, and **c** pEGFP-N2-PDGFR-β-EPCs group. Arrows indicate TUNEL-positive cells (green). Red: VSMCs stained with SmαA; Blue: nuclei stained with DAPI. **d** TUNEL-labeling index. **P < 0.01 vs. saline control group; ^##^ *P* < 0.01 vs. pEGFP-N2-EPCs group (*n* = 5). L, lumen; M, media. Scale bar = 50 μm

We next examined the effects of transplantation of EPCs overexpressing PDGFR-β on intima cells apoptosis/necrosis by TUNEL staining (Fig. 7a,b and c). After 7 days of the carotid injury, the TUNEL-labeling index was significantly greater in the pEGFP-N2-PDGFR-β-EPCs group (36.45 ± 5.83) than in the pEGFP-N2-EPCs group (24.45 ± 6.08, *p* < 0.01, Fig. 7d), indicating increased intima cell apoptosis was associated with PDGFR-β overexpression.

### Effects of EPCs overexpressing PDGFR-β on PDGF-BB-induced VSMC migration

**Fig. 8:**
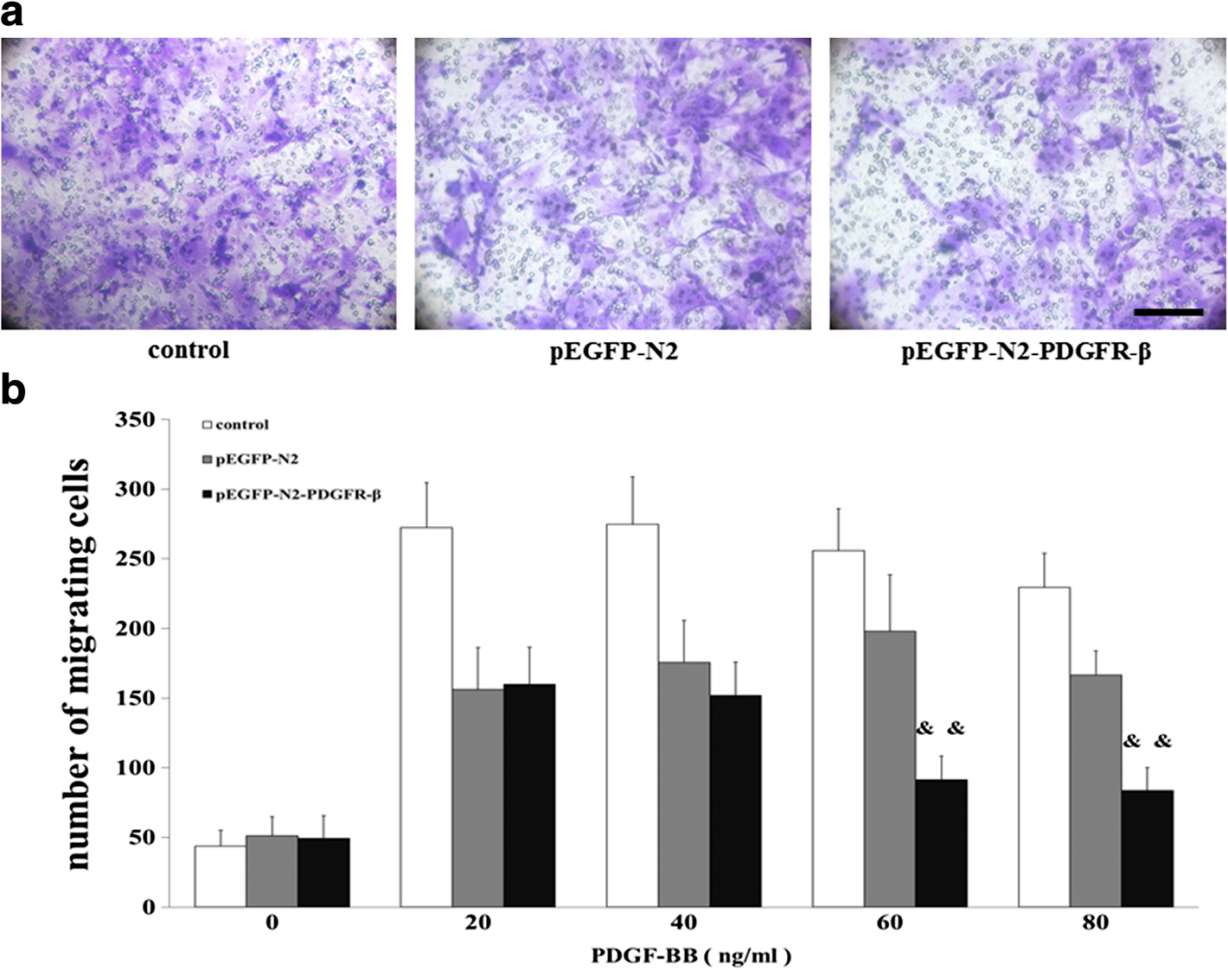
Effects of PDGF-BB induced VSMC migration. **a** Representative images from VSMCs migration by recombinant PDGF-BB occurred at 60 ng/mL. **b** VSMC migration was examined by the co-culture migration assay.&& *P* < 0.01 vs. pEGFP-N2-PDGFR-β cells under 0 ng/mL PDGF-BB stimulation. Scale bar = 100 μm

To examine the effects of PDGF-BB-induced VSMCs, co-culture Transwell system was used. The main effect of concentration (F = 271.088, *P* < 0.01) and group (F = 335.35, *P* < 0.01), as well as their interaction (F = 34.699, *P* < 0.01), were all significant. The maximum migration induced by recombinant PDGF-BB occurred at 20 ng/mL in the control group and pEGFP-N2-PDGFR-β groups and at 60 ng/mL in the pEGFP-N2 group (Fig. 8b). Interestingly, in the pEGFP-N2-PDGFR-β group, VSMCs migration decreased significantly with increase in PDGF-BB concentration (*P* < 0.01) (Fig. 8a, b), indicating that PDGF-BB-induced VSMCs migration is attenuated by EPCs overexpressing PDGFR-β.

### Effects of EPCs overexpressing PDGFR-β on PDGF-BB treatments reduced VSMCs apoptosis

**Fig. 9:**
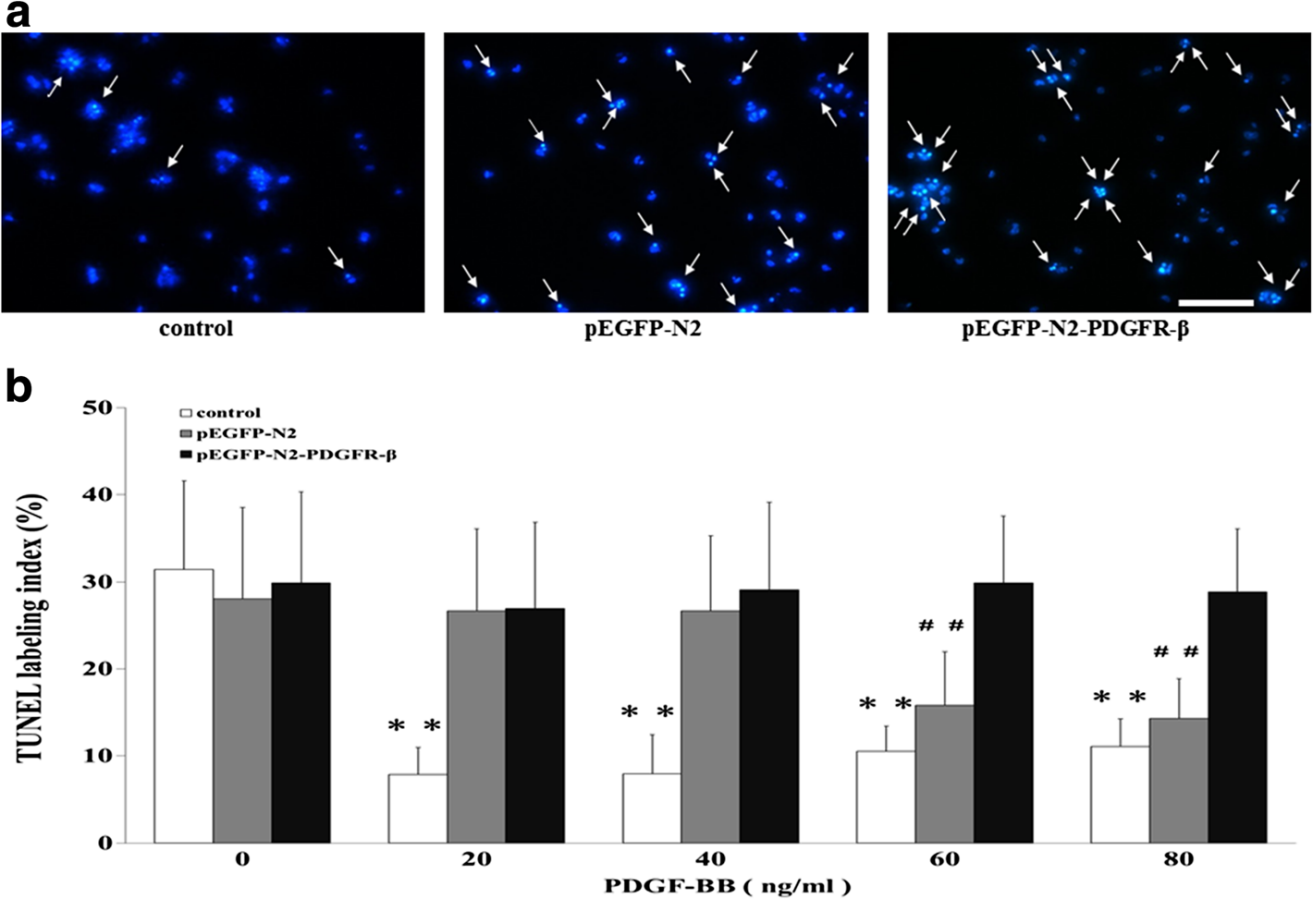
Effects of PDGF-BB treatments reduced VSMC apoptosis. **a** Representative images from VSMCs apoptosis when recombinant PDGF-BB occurred at 80 ng/mL. Arrows indicate TUNEL-positive cells (green). **b** VSMC apoptosis was examined by using the in situ Cell Death Detection Kit. ** *P* < 0.01 vs. Control cells under 0 ng/mL PDGF-BB stimulation. ^##^ *P* < 0.01 vs. pEGFP-N2 cells under 0 ng/mL PDGF-BB stimulation. Scale bar = 100 μm

VSMCs apoptosis plays an important role in vascular remodeling. We then used the in situ Cell Death Detection Kit to examine the effects of PDGF-BB treatments reduced VSMCs apoptosis. The main effects of concentration (F = 17.798, *P* < 0.01) and group (F = 74.428, *P* < 0.01), as well as their interaction (F = 10.376, *P* < 0.01), were all significant. The minimum TUNEL-labeling index was at 20 ng/mL PDGF-BB in the control group and at 80 ng/mL PDGF-BB in the pEGFP-N2 group respectively (Fig. 9a). In contrast, in the pEGFP-N2-PDGFR-β group, TUNEL-labeling index remained unchanged under different concentrations of PDGF-BB (*P* > 0.05) (Fig. 9b), indicating that PDGF-BB treatments reduced VSMCs apoptosis is attenuated by EPCs overexpressing PDGFR-β.

## Discussion

In this study, we found that transplantation of EPCs overexpressing PDGFR-β significantly promoted reendothelialization in the early phase after carotid artery injury in mice. Additionally, EPCs overexpressing PDGFR-β inhibited neointima formation via increasing apoptosis and suppressing proliferation of VSMCs. The cause of limited neointima formation might be that homed EPCs overexpressing PDGFR-β can compete for the locally produced PDGF-BB which is a result of injured arteries with the VSMCs, therefore inhibiting the local VSMC migration, proliferation, antiapoptotic function, and reduced neointima formation. This assumption then confirmed by our experimental results in vitro. This study first reported that transplantation of genetically modified EPC can have a combined effect of both amplifying the reendothelialization capacity of EPCs and inhibiting neointima formation so as to facilitate better inhibition of adverse remodeling after vascular injury.

EPCs can differentiate into mature ECs in vivo and therefore play an important role in vascular endothelial repair and angiogenesis in the ischemic tissues [17, 20–23]. The number and biological function of the body’s circulating EPCs largely reflect the ability of vascular repair and microvascular reconstruction of the ischemic tissues [6, 8, 24, 25]. In certain diseases such as diabetes, coronary heart disease, and chronic renal failure, the number and/or biological function of the circulating EPCs decreases significantly, causing and/or facilitating the occurrence and development of these diseases [6, 8, 24–26]. Even under the normal physiological condition, in case of acute vascular injury or acute tissue ischemia (e.g., vascular endothelial damage due to a surgery), short-time blocking of the blood flow to organs, and shock leading to systemic perfusion, the number of circulating EPCs in the body are insufficient for the repair of the damaged vascular endothelium, for remodeling of the microvascular network, and for angiogenesis [17, 21, 27–29].

Previous studies have confirmed that EPC mobilization or transplantation to increase the number of circulating EPCs can promote the repair of damaged vascular endothelium [17, 21, 22, 30–36]. Transplantation of EPCs, whose biological functions have been improved by genetic modification or cytokine induction, could further enhance the repair of damaged blood vessels, as observed in some previous studies [37, 38]. In addition, a few studies reported that applying the receptor–ligand interaction mechanism to promote directional chemotaxis of EPCs could increase the number of EPCs homing to the damaged blood vessels [16, 38–40]. Both these approaches, acting on a single link, can achieve a certain effect, but not the desired effect. Presently, the homing, proliferation, and differentiation of circulating EPCs as well as the interaction of these cells with the surrounding tissue cells remain to be investigated.

Recent data have shown that remote ischaemic preconditioning reduced the incidence of periprocedural myocardial infarction following PCI [41]. Vascular interventional therapy as well as several other factors such as blood flow stress can lead to vascular intima damage, which can result in adhesion and aggregation of a large number of platelets in the intimal lesion. The adhesion of platelets releases a large amount of PDGF. Meanwhile, vascular intimal injury induces transformation of local VSMCs from contractile cells to secretory cells, leading to synthesis and secretion of a large amount of PDGF [42, 43]. PDGF exerts biological activity through the paracrine and/or autocrine glands. The PDGF family, especially PDGF-BB, is closely involved with the restenosis of the target vessels after PCI, atherosclerosis, and other vascular intimal proliferative diseases. Under normal physiological conditions, the normal artery walls have very low PDGF-BB expression level, but the PDGF-BB expression level in the target vascular tissue after PCI or in atherosclerotic vascular lesions is increased [44–46].

The local production and release of PDGF-BB exert its function in an autocrine and/or paracrine manner on the local VSMCs after vascular injury. It promotes proliferation, migration, and phenotype transformation of VSMCs [47]. In addition, it promotes postoperative restenosis after PCI and the formation of atherosclerosis plate [14, 48]. A recent study has also shown that PDGF-BB can induce proliferation, migration, and angiogenesis of EPCs over-expressing PDGFR- β receptor [16].

PDGF-BB functions mainly by acting on its specific receptor. PDGFR is a transmembrane glycoprotein possessing protein tyrosine kinase activity composed of two subunits, alpha and beta. The beta subunit PDGFR-β plays an important role in the occurrence and development of vascular intimal proliferative diseases induced by PDGF-BB [14, 48]. PDGFR-β-specific receptor blockers can effectively inhibit neointimal hyperplasia induced by PDGF-BB and reduce the degree of stenosis after vascular injury [49, 50].

Our result is consistent with previous report that the local expression and release of PDGF-BB increased during the acute period after carotid artery injury in mice and that this increase was sustained for a long time (approximately 2 weeks) [51, 52]. After transplantation of EPCs overexpressing PDGFR-β or EPCs with blank plasmid, we found that the number of EPCs homing to the injured arteries was significantly higher in the PDGFR-β overexpression group than in the PDGFR-β non-overexpression group (blank plasmid-transfected EPCs group). Evans Blue staining revealed that the reendothelialization area of injured carotid arteries was significantly higher in the PDGFR-β overexpression group than in the PDGFR-β non-overexpression group at both day 7 and day 14 and improved reendothelialization was more dramatic at day 14 compared to day 7.

Reendothelialization at sites of spontaneous or iatrogenic disruption has classically been thought to be a result from the migration and proliferation of ECs from viable endothelium adjacent to the sites of injury. Circulating EPCs as optimal candidates in cell-based therapies for vascular diseases has been well documented in contributing to the maintenance of endothelial integrity, function, and regeneration of injured endothelium [7–10]. The number, migratory capacity, and proliferative capacity of circulating EPCs are the main factors determine their ability to home to and incorporate into sites of reendothelialization. Our previous study has shown that a stably high expression of PDGFR-β of EPCs can be achieved by transfecting EPCs with pEGFP-N2-PDGFR-β. The stimulus of exogenous PDGF-BB can significantly enhance the capability of proliferation, migration, and angiogenesis in EPCs overexpressing PDGFR-β in vitro [16]. These could explain why both the number of EPCs homing to the injured arteries and the reendothelialization area of injured carotid arteries were significantly higher in the PDGFR-β overexpression group than in the PDGFR-β non-overexpression group.

We further asked whether increased reendothelialization has an impact on neointima formation. We performed HE staining and found the inhibition of neointima formation at day 14 after arterial injury in the PDGFR-β overexpression group. This result suggests that PDGFR-β mediated reendothelialization is reversely correlated with neointima formation during vascular regeneration at the injury site. To seek the cause of decreased neointima formation, we further performed TUNEL staining to evaluate cell apoptosis to analyze inhibition of neointimal hyperplasia by transplanted EPCs overexpressing PDGFR-β after arterial injury. We found that transplanted EPCs with PDGFR-β overexpression can promote local VSMCs apoptosis in the injured carotid artery in mice at day 7 after cell transplantation. Then, we established the VSMC/EPC co-culture system in vitro. Our data shown that PDGF-BB-induced VSMC migration and PDGF-BB treatments VSMC antiapoptotic function is attenuated by EPCs overexpressing PDGFR-β competitively consume the PDGF-BB.

There are some limitations in our paper: First, our observations are based on a relatively simple animal model (young and healthy mice), and thus, the study conclusions may be limited to non-atherosclerotic arteries. Second, we did not observe the enhancing effects of the endogenous PDGF-BB, released locally by the injured carotid arteries in mice, on the recruitment of EPCs over-expressing PDGFR-β. The above-mentioned limitations would be addressed in our future studies.

## Conclusions

Our present study suggests that the interaction between the transplanted EPCs overexpressing PDGFR-β and the PDGF-BB expressed and released locally by the injured carotid arteries of mice can promote homing of EPCs overexpressing PDGFR-β to the injured arteries, accelerate reendothelialization of the injured artery, and inhibit neointimal proliferation of the injured arteries after vascular injury. In addition, overexpression of PDGFR-β in the recruited EPCs can competitively consume the PDGF-BB generated locally by the injured arteries, promoting proliferation, migration, and anti-apoptosis of vascular VSMCs, which in turn can strengthen the inhibition of neointimal hyperplasia induced after vascular injury. Thus, our results suggest that the transplantation of EPCs overexpressing PDGFR-β can be used as a novel therapeutic approach for the treatment of vascular injury diseases.

## Abbreviations

ANOVA: Analysis of variance
DMEM/F-12: Dulbecco’s modified eagle medium: nutrient mixture F-12
ECs: Endothelial cells
EGFP: Enhanced green fluorescent protein
EPCs: Endothelial progenitor cells
FCS: Fetal calf serum
HE: Hematoxylin and eosin
HPFs: High-power fields
IP: Iintraperitoneal
NI/M: Neointimal /media
OCT: Optimal cutting temperature
PDGFR: Platelet-derived growth factor receptor
PO: Per os
PVDF: Polyvinylidene fluoride
RT-PCR: Reverse transcription-polymerase chain reaction
SMαA: Smooth muscle α-actin
TUNEL: Transferase dUTP nick-end labeling
VSMCs: Vascular smooth muscle cells
